# Sex‐Specific Association Between Childhood Adversity and Accelerated Biological Aging

**DOI:** 10.1002/advs.202309346

**Published:** 2024-05-05

**Authors:** Jie Yu, Fan Pu, Gan Yang, Meng Hao, Hui Zhang, Jingyun Zhang, Xingqi Cao, Lijun Zhu, Yuhui Wan, Xiaofeng Wang, Zuyun Liu

**Affiliations:** ^1^ Center for Clinical Big Data and Analytics of the Second Affiliated Hospital and Department of Big Data in Health Science School of Public Health The Key Laboratory of Intelligent Preventive Medicine of Zhejiang Province Zhejiang University School of Medicine Hangzhou 310058 China; ^2^ Human Phenome Institute and State Key Laboratory of Genetic Engineering Zhangjiang Fudan International Innovation Center School of Life Sciences Fudan University Shanghai 200433 China; ^3^ National Clinical Research Center for Ageing and Medicine Huashan Hospital Fudan University Shanghai 200433 China; ^4^ Zhejiang Provincial Key Laboratory for Diagnosis and Treatment of Aging and Physic‐chemical Injury Diseases The First Affiliated Hospital Zhejiang University School of Medicine Hangzhou 310058 China; ^5^ MOE Key Laboratory of Population Health across Life Cycle/Anhui Provincial Key Laboratory of Population Health and Aristogenics and Department of Maternal Child and Adolescent Health School of Public Health Anhui Medical University Hefei 230032 China

**Keywords:** aging, childhood adversity, lifestyle, mental disorder, sex difference, telomere length

## Abstract

Is childhood adversity associated with biological aging, and if so, does sex modify the association, and do lifestyle and mental health mediate the association? A lifespan analysis is conducted using data on 142 872 participants from the UK Biobank to address these questions. Childhood adversity is assessed through the online mental health questionnaire (2016), including physical neglect, physical abuse, emotional neglect, emotional abuse, sexual abuse, and a cumulative score. Biological aging is indicated by telomere length (TL) measured from leukocyte DNA using qPCR, and the shorter TL indicates accelerated biological aging; a lifestyle score is constructed using body mass index, physical activity, drinking, smoking, and diet; mental disorder is assessed using depression, anxiety, and insomnia at the baseline survey. The results reveal a sex‐specific association such that childhood adversity is associated with shorter TL in women after adjusting for covariates including polygenic risk score for TL, but not in men. Unhealthy lifestyle and mental disorder partially mediate the association in women. The proportions of indirect effects are largest for sexual and physical abuse. These findings highlight the importance of behavioral and psychological interventions in promoting healthy aging among women who experienced childhood adversity, particularly sexual and physical abuse.

## Graphical abstract

Childhood adversity is associated with shorter telomere length, an indicator of accelerated biological aging, in women after adjusting for polygenic risk score for telomere length, but not in men. Unhealthy lifestyle and mental disorder partially mediate the association in women. The proportions of indirect effects are largest for sexual and physical abuse.

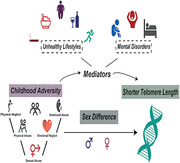

## 1 Introduction

Aging, a complicated multisystemic process, does not always approach steadily and chronologically. Accelerated aging is an acknowledged risk factor for various chronic diseases and mortality.^[^ ^1^ ^]^ Among the various aging indicators, telomere length (TL) is a widely used and reliable one. Telomeres are DNA‐protein complexes at the ends of chromosomes providing genomic stability.^[^ ^2^ ^]^ TL shortening seems to reflect the accumulated burden of oxidative stress related to aging^[^ ^3^, ^4^ ^]^ and increased risk of aging related diseases and mortality.^[^ ^5^ ^]^ Except for innate factors (inherited genetic variation [single nucleotide polymorphisms (SNPs)]),^[^ ^6^, ^7^ ^]^ ascertaining life course exposures associated with TL may help plan more precise and cost‐effective policies to relieve the burden of aging related diseases.^[^ ^8^, ^9^ ^]^

Recently, several studies have investigated the association between childhood adversity and TL but yielded conflicting findings,^[^ ^10^ ^]^ including negative associations,^[^ ^11^, ^12^, ^13^ ^]^ positive associations,^[^ ^14^ ^]^ and non‐significant results.^[^ ^15^, ^16^ ^]^ One possible explanation could be attributed to the population structure in terms of sex; such that the inherent sex difference in physical and mental aspects may bias the findings. On the one hand, men are more likely to report physical abuse, whereas women are more likely to report sexual abuse, emotional abuse, and neglect, as well as a greater number of adversities.^[^ ^17^, ^18^, ^19^ ^]^ On the other hand, different gonadal hormone changes that occur over dynamic periods of development and maturation cause sex differences in the hypothalamic‐pituitary‐adrenal (HPA) stress axis (e.g., the relative size and steroidogenic activity of the adrenal gland cortex), contributing to sex‐specific stress responses and vulnerabilities across the lifespan.^[^ ^20^, ^21^ ^]^ However, few studies have clarified the possible sex difference in the association between childhood adversity and TL.^[^ ^10^ ^]^ Next, disentangling the question of how childhood adversity casts a distal influence on TL in later life is of interest. According to the biopsychosocial model first conceptualized by George Engel in 1977,^[^ ^22^ ^]^ psychological and social factors have drawn much attention beyond the biological mechanisms, partially due to that both of them are modifiable, providing a window for intervention. Given that poor lifestyles^[^ ^23^, ^24^ ^]^ and mental disorders^[^ ^24^, ^25^ ^]^ have been related to psychosocial stress and accelerated aging, we hypothesize that these factors might mediate the possible negative association between childhood adversity and TL.

To fill these knowledge gaps, we used a large sample of 142 872 adults from the UK Biobank (UKB) and aimed to examine 1) whether childhood adversity was associated with TL; 2) whether sex modified the association; and 3) whether lifestyle and mental disorder mediated the association as potential mechanisms. We validated the results with datasets available in US Health and Retirement Study (HRS).

## 2 Results

### 2.1 Characteristics of the Study Participants

The characteristics of the participants are presented in **Table** **1**. The mean ± standard deviation (SD) of age was 56.4 ± 7.7 years; 80 298 participants (56.2%) were women, and 138 945 (97.3%) were White. Compared with men, women had a higher cumulative score (0.9 ± 1.2 vs 0.8 ± 1.0) and a higher percentage for individual types of childhood adversity except for physical abuse. TL was longer in women than that in men (0.85 ± 0.13 vs 0.82 ± 0.13).

**Table 1:** Characteristics of the study participants.

	Total (N = 142 872)	Women (N = 80 298)	Men (N = 62 574)	P value
Age, mean (SD), y	56.4 (7.7)	55.9 (7.6)	57.0 (7.8)	<.001
Race and ethnicity				<.001
White	138 945 (97.3)	78 067 (97.2)	60 878 (97.3)	
Chinese	323 (0.2)	228 (0.3)	95 (0.2)	
South Asian	1146 (0.8)	513 (0.6)	633 (1.0)	
Black	948 (0.7)	547 (0.7)	401 (0.6)	
Multiple	733 (0.5)	466 (0.6)	267 (0.4)	
Other^a^	777 (0.5)	477 (0.6)	300 (0.5)	
Educational level^b^				<.001
High	66 134 (46.3)	36 124 (45.0)	30 010 (48.0)	
Intermediate	47 064 (32.9)	28 664 (35.7)	18 400 (29.4)	
Low	29 674 (20.8)	15 510 (19.3)	14 164 (22.6)	
Occupation				<.001
Working	91 154 (63.8)	50 441 (62.8)	40 713 (65.1)	
Retired	42 278 (29.6)	23 269 (29.0)	19 009 (30.4)	
Other^c^	9440 (6.6)	6588 (8.2)	2852 (4.6)	
Townsend deprivation index, mean (SD)	−1.7 (2.8)	−1.7 (2.8)	−1.8 (2.8)	<.001
Depression, yes	7537 (5.3)	5162 (6.4)	2375 (3.8)	<.001
Anxiety, yes	2119 (1.5)	1364 (1.7)	755 (1.2)	<.001
Insomnia, yes	37 262 (26.1)	23 434 (29.2)	13 828 (22.1)	<.001
BMI, mean (SD), kg/m^2^	26.8 (4.6)	26.3 (4.9)	27.3 (4.0)	<.001
≤18.5	793 (0.6)	677 (0.8)	116 (0.2)	
18.5‐24.9	54 648 (38.3)	36 348 (45.4)	18 300 (29.3)	
≥24.9	87 138 (61.1)	43 111 (53.8)	44 027 (70.5)	
Smoking, yes	59 974 (42.0)	29 848 (37.2)	30 126 (48.1)	<.001
Drinking, yes	56 844 (39.8)	34 349 (42.8)	22 495 (35.9)	<.001
Irregular exercise, yes	36 746 (25.7)	21 547 (26.8)	15 199 (24.3)	<.001
Unhealthy diet, yes	87 725 (61.4)	46 962 (58.5)	40 763 (65.1)	<.001
Prevalent CVD, yes	6291 (4.4)	2048 (2.6)	4243 (6.8)	<.001
Prevalent cancer, yes	11 086 (7.8)	7280 (9.1)	3806 (6.1)	<.001
Polygenic risk score for telomere length, mean (SD)	−0.42 (0.11)	−0.42 (0.11)	−0.42 (0.11)	0.550
Childhood adversity, yes				
Physical neglect	22 968 (16.1)	13 544 (16.9)	9424 (15.1)	<.001
Emotional neglect	31 496 (22.0)	18 170 (22.6)	13 326 (21.3)	<.001
Sexual abuse	12 533 (8.8)	8881 (11.1)	3652 (5.8)	<.001
Physical abuse	26 785 (18.7)	13 673 (17.0)	13 112 (21.0)	<.001
Emotional abuse	21 921 (15.3)	14 043 (17.5)	7878 (12.6)	<.001
Cumulative score	0.8 (1.1)	0.9 (1.2)	0.8 (1.0)	<.001
Telomere length, mean (SD)	0.84 (0.13)	0.85 (0.13)	0.82 (0.13)	<.001

Abbreviation: BMI, body mass index; CVD, cardiovascular disease. Data are presented as means (SD) for continuous variables and numbers (percentage) for categorical variables. *P*‐values were calculated using t‐test for continuous variables and χ^2^ test for categorical variables;

^a)^ Other includes any races or ethnicities not otherwise specified

^b)^ High educational level: college or university degree; Intermediate educational level: A/AS levels or equivalent, O levels/GCSEs or equivalent; Low educational level: none of the aforementioned;

^c)^ Other includes full‐time or part‐time student, unpaid or voluntary work, unemployed, unable to work because of sickness or disability, looking after home and/or family, and did not answer.

### 2.2 Association of Childhood Adversity with Telomere Length (TL) in All Participants

As shown in **Table** **2**, childhood adversity was significantly associated with TL. A higher cumulative score was associated with shorter TL. We observed dose‐response associations of the cumulative score with TL (*P* for trend < 0.001). In model 2, participants experiencing four (β = −0.0067; 95% confidence intervals [CI]: −0.0108, −0.0026) or five (β = −0.0101; 95% CI: −0.0173, −0.0028) childhood adversities had shorter TL, compared with those who did not experience childhood adversity. Among all childhood adversities, physical and sexual abuse substantially contributed to the TL shortening, whereas emotional adversities were less noticeable. For instance, in model 2, participants who experienced physical (β = −0.0045; 95% CI: −0.0063, −0.0028) or sexual abuse (β = −0.0035; 95% CI: −0.0059, −0.0011) had a shorter TL compared with those who did not experience. After further adjustment for polygenic risk score (PRS) for TL, these associations were unchanged (Model 3).

**Table 2:** Associations of childhood adversity with telomere length in all participants (N = 142872).

Childhood adversity	Model 1^a^		Model 2^b^		Model 3^c^	
	β (95% CI)	*P* value	β (95% CI)	*P* value	β (95% CI)	*P* value
Physical neglect	−0.0027 (−0.0046, −0.0009)	0.003	−0.0024 (−0.0043, −0.0006)	0.010	−0.0024 (−0.0043, −0.0006)	0.010
Emotional neglect	−0.0007 (−0.0024, 0.0009)	0.367	−0.0008 (−0.0024, 0.0008)	0.319	−0.0008 (−0.0024, 0.0008)	0.326
Sexual abuse	−0.0029 (−0.0053, −0.0005)	0.018	−0.0035 (−0.0059, −0.0011)	0.004	−0.0033 (−0.0057, −0.0009)	0.007
Physical abuse	−0.0040 (−0.0058, −0.0023)	<.001	−0.0045 (−0.0063, −0.0028)	<.001	−0.0045 (−0.0062, −0.0028)	<.001
Emotional abuse	−0.0019 (−0.0037, 0.0000)	0.050	−0.0023 (−0.0042, −0.0004)	0.016	−0.0022 (−0.0040, −0.0003)	0.023
Cumulative score	−0.0013 (−0.0019, −0.0007)	<.001	−0.0014 (−0.0020, −0.0008)	<.001	−0.0014 (−0.0020, −0.0008)	<.001
0	Ref.		Ref.		Ref.	
1	0.0003 (−0.0014, 0.0019)	0.759	0.0002 (−0.0014, 0.0018)	0.803	0.0002 (−0.0014, 0.0018)	0.808
2	−0.0020 (−0.0042, 0.0002)	0.073	−0.0022 (−0.0044, 0.0000)	0.047	−0.0023 (−0.0045, −0.0001)	0.040
3	−0.0036 (−0.0065, −0.0006)	0.018	−0.0040 (−0.0069, −0.0010)	0.009	−0.0039 (−0.0068, −0.0009)	0.010
4	−0.0062 (−0.0103, −0.0021)	0.003	−0.0067 (−0.0108, −0.0026)	0.001	−0.0062 (−0.0103, −0.0021)	0.003
5	−0.0088 (−0.0161, −0.0016)	0.017	−0.0101 (−0.0173, −0.0028)	0.007	−0.0102 (−0.0174, −0.0029)	0.006
*P* for trend	<.001		<.001		<.001	

Abbreviation: CI, confidence interval. *P* values were calculated using general linear regression models;

^a)^ Model 1 was adjusted for age and sex;

^b)^ Model 2 was further adjusted for race and ethnicity, educational level, occupation, Townsend deprivation index and history of cardiovascular disease and cancer based on Model 1;

^c)^ Model 3 was further adjusted for polygenic risk score for telomere length based on Model 2.

### 2.3 Sex Differences in the Association of Childhood Adversity with TL

**Figure 1:**
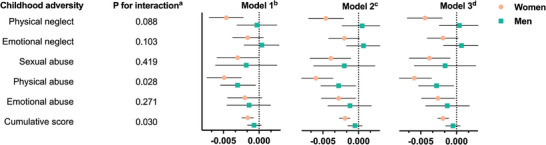
Associations of childhood adversity with telomere length by sex (N = 142872). *P* values were calculated using general linear regression models. ^a)^ The β coefficients of interaction terms were −0.0032 for physical neglect, −0.0027 for emotional neglect, −0.0021 for sexual abuse, −0.0038 for physical abuse, −0.0022 for emotional abuse, and −0.0014 for cumulative score. Women and men were coded as 1 and 0, respectively. ^b)^ Model 1 was adjusted for age. ^c)^ Model 2 was further adjusted for race and ethnicity, educational level, occupation, Townsend deprivation index and history of cardiovascular disease and cancer based on Model 1. ^d)^ Model 3 was further adjusted for polygenic risk score for telomere length based on Model 2.

**Figure** **1** shows the associations of childhood adversity with TL stratified by sex. In the fully adjusted model, both the cumulative score and individual type of childhood adversity were associated with shorter TL in women, while only physical abuse was significantly associated with TL in men. Significant interactions of cumulative score (*P* = 0.030) and physical abuse (*P* = 0.028) with sex on shorter TL were observed.

### 2.4 Mediation Analyses of Unhealthy Lifestyle Index and Mental Disorder Index in the Association of Childhood Adversity with TL in Women

**Figure 2:**
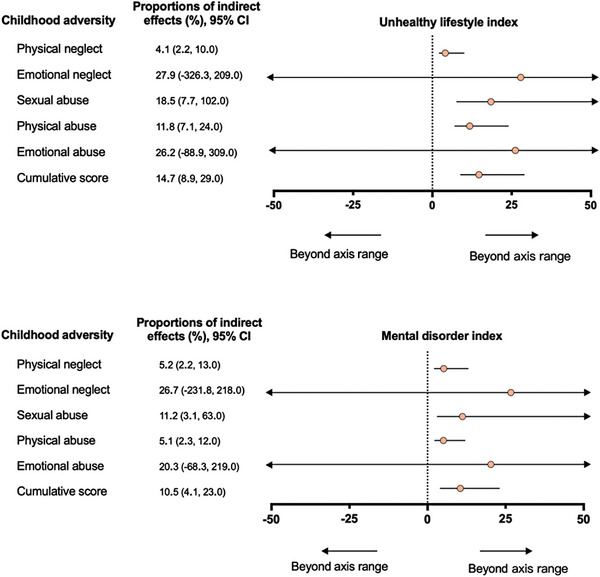
Proportions of indirect effects of childhood adversity in telomere length attributed to unhealthy lifestyle index and mental disorder index in women (N = 80298). Abbreviation: CI, confidence interval. All models were adjusted for age. Proportions of indirect effects were calculated by mediation analysis using R package “mediation”.

First, Tables S1 and S2 (Supporting Information) show the associations of childhood adversity with unhealthy lifestyle index and mental disorder index in women. In the fully adjusted model (Model 2), both the individual type of childhood adversity and cumulative score were associated with higher unhealthy lifestyle scores and mental disorder scores in women. Second, Tables S3 and S4 (Supporting Information) show that higher unhealthy lifestyle scores and mental disorder scores were associated with shorter TL in women in model 2. Finally, **Figure** **2** shows the proportions of indirect effects of childhood adversity in TL attributed to unhealthy lifestyle index and mental disorder index. Unhealthy lifestyle index partially mediated 4.1% to 18.5% in the associations of cumulative score, physical abuse, and sexual abuse with TL in women. The proportions of indirect effects were largest for sexual (18.5%) and physical (11.8%) abuse. Mental disorder index partially mediated 5.1% to 11.2% in the associations of cumulative score, physical abuse, physical neglect, and sexual abuse with TL in women. The proportion of indirect effect was largest for sexual abuse (11.2%).

### 2.5 Additional Analyses

First, compared with the included participants, we found that participants excluded due to missing data were less likely to be White, more likely to be women and had a lower Townsend Deprivation Index (TDI) (Table S5, Supporting Information). Second, sensitivity analysis showed similar results when using a complete‐case sample (N = 66492) (Table S6, Supporting Information).

### 2.6 Replication in the US Health and Retirement Study

**Figure 3:**
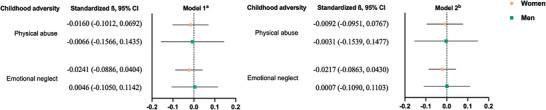
Associations of childhood adversity with telomere length in HRS (N = 5282). Abbreviation: CI, confidence interval. β (95% CI) were calculated using general linear regression models. ^a)^ Model 1 was adjusted for age. ^b)^ Model 2 was further adjusted for race and ethnicity, educational level, marital status, history of smoking, body mass index, and number of medical conditions based on Model 1.

Among all types of childhood adversity in the UKB, only physical abuse and emotional neglect could be mimicked in US HRS (N = 5282). The characteristics of the participants are presented in Table S7 (Supporting Information). Compared with men, women had a higher percentage for both physical abuse (8.6% vs 5.2%) and emotional neglect (16.4% vs 10.3%). In general linear regression models, the differences between men and women were similarly observed as to UKB (**Figure** **3**). For example, in the fully adjusted model, the standardized coefficients for emotional neglect were −0.0217 in women and 0.0007 in men, although it was not statistically significant probably due to the relatively small sample size.

## 3 Discussion

In this large lifespan analysis of middle‐aged and older adults in the UKB, we found a sex‐specific association between childhood adversity and shorter TL. Nearly all types of childhood adversity were associated with shorter TL in women but not in men. We further explored the role of social and mental factors in the association based on the biopsychosocial model^[^ ^22^ ^]^ and found that unhealthy lifestyle and mental disorder partially mediated the link between childhood adversity and shorter TL in women. Moreover, physical and sexual abuse substantially contributed to the TL shortening through unhealthy lifestyle and mental disorder. These findings highlight the potential of behavioral and psychological interventions on childhood adversity to slow biological aging and thus diminish health inequalities in later life. Furthermore, special attention should be given to women who experienced physical and sexual abuse.

In addition to having observed the significant association of childhood adversity with TL in the full sample as done in previous studies,^[^ ^11^, ^12^ ^]^ this study is the first to clearly and comprehensively address sex differences in a large sample of adults. The sex differences could be explained in several aspects. First, men and women tend to report different types and numbers of childhood adversity. Women are more inclined to report sexual and emotional adversities than men.^[^ ^18^ ^]^ Second, the stress responses and vulnerabilities are sex‐specific due to the differences in the HPA stress axis caused by different gonadal hormone changes.^[^ ^20^ ^]^ Third, women have longer TL than men,^[^ ^26^ ^]^ possibly because estrogen may stimulate telomerase to add telomeres to the ends of chromosomes, thereby extending TL.^[^ ^27^ ^]^ Besides, telomerase activity is higher in women embryo cells, which could result in longer TL in women prior to embryo implantation, at birth, and even throughout life.^[^ ^28^ ^]^ However, our study warned that even though TL is longer in women, the risk of childhood adversity on biological aging is much higher in women than in men and should not be ignored.

We further demonstrated that childhood adversity was associated with TL shortening partially through behavior and mental disorder in women, supporting the biopsychosocial model conceptualized by George Engel.^[^ ^22^ ^]^ Previous studies have indicated that childhood adversity may lead to the adoption of unhealthy lifestyle to reduce stress, which may accelerate aging in the long term.^[^ ^23^ ^]^ Our study further found that women with childhood adversity were more susceptible to adopting unhealthy lifestyle than men, suggesting that emphasis should be laid on women's lifestyle intervention. Besides, early negative psychological events (e.g., maladaptive schema, avoidance, and impaired resilience) caused by childhood adversity may activate the HPA axis, resulting in the sensitization of depression pathways,^[^ ^29^, ^30^ ^]^ which may have a distal influence on oxidative stress, immune system, and metabolic abnormalities, consequently driving TL shortening and biological aging.^[^ ^31^, ^32^ ^]^ Previous studies also found that the associations between traumatic experience and depressive and anxiety symptoms were larger in women.^[^ ^33^, ^34^, ^35^ ^]^ Similarly, emerging evidence have suggested that childhood adversity increased the risk of multiple sleep disorders including insomnia, particularly sexual abuse in women.^[^ ^36^ ^]^ In short, our study adds to the field by illustrating the proportion of the total effect mediated through unhealthy lifestyle and mental disorder in women.

Another important finding in our study is that among all childhood adversities, physical and sexual abuse substantially contributed to the TL shortening, compared with emotional adversities. In men, only physical abuse was associated with shorter TL. In women, those who experienced both were more likely to accelerate aging possibly by triggering an unhealthy lifestyle and mental disorder. Evidence shows that childhood abuse could result in a series of health problems through behavioral, social, emotional, and cognitive pathways,^[^ ^37^ ^]^ especially in women.^[^ ^38^ ^]^ For instance, physical and sexual abuse have consistently been associated with accelerated pubertal development in women, which is also a metric of biological aging, while existing studies typically found no association of emotional neglect with pubertal timing.^[^ ^39^ ^]^ It is worth noting that the percentage of sexual abuse was much higher among women than among men (11.1% vs 5.8%). Moreover, previous studies indicated that women with childhood sexual abuse had a higher risk for major depression,^[^ ^40^ ^]^ cancer,^[^ ^41^ ^]^ and reduced self‐perceived general health.^[^ ^42^ ^]^ These findings revealed that the associations of childhood adversity with shorter TL might vary according to the distinct types of the adversity experienced. Thus, it is necessary to further strengthen the monitoring of physical and sexual abuse in childhood and provide targeted interventions for those who suffered from physical and sexual abuse.

Our findings have important implications for public health. First, the sex differences in the association of childhood adversity with biological aging suggest that boys and girls warrant different attention and policies by society and the government. Extra efforts might be needed to develop avenues for breaking the transmission of childhood adversity in girls. Second, the partial indirect effects of unhealthy lifestyle and mental disorder in the association suggest that behavioral and psychological interventions would probably help to alleviate the toll of childhood adversity on women and promote healthy aging. Previous randomized controlled trials have confirmed that cognitive‐behavioral therapy and child‐parent psychotherapy were effective in improving outcomes (e.g., depression, post‐traumatic stress disorder, anxiety, and sexual problems) for children exposed to childhood adversity.^[^ ^43^, ^44^ ^]^ Third, the interventions should target physical and sexual abuse in girls, which could be identifiable and modifiable. The affected children often show early indications of bodily injury and poor academic performance.^[^ ^45^ ^]^ Thus, there is a compelling need for early detection of these indications and timely interventions by health services and schools. For instance, trained health‐care providers should identify vulnerable children and offer specialized psychological counseling for their traumas. For schools, ensuring the social inclusion and rectifying unhealthy lifestyle for the affected children might be needed to prevent lifelong diseases.

Major strengths of our study included the large population of middle‐aged and older adults and the additional adjustment for genetic susceptibility (measured by PRS) in the models. Moreover, we deeply explored the underlying mechanisms of the association of childhood adversity with TL from behavioral and psychological perspectives. This study also has several limitations. First, the assessment of childhood adversity in this study lacked details, such as the duration and severity of childhood adversity. Therefore, more studies are needed to further enrich the measure of childhood adversity to reinforce our findings. Second, information on childhood adversity was self‐reported in 2016, which may result in age‐dependent recall bias. For instance, some adults with extremely negative childhood experiences may exaggerate their adversities and overestimate the impact of adversities, whereas more agreeable dispositions may bias adversity measures toward underestimating the impact of adversity on TL.^[^ ^46^ ^]^ Third, men may tend to hide parts of their childhood adversity when reporting, while women are more likely to speak out, which may lead to an inconsistency with the actual number of adversities. The effect of childhood adversity on TL may be underestimated in men. Fourth, absence of detecting outcome variable (TL) in childhood as well as the mediators (unhealthy lifestyle and mental disorder) may result in uncertain causal relationship between childhood adversity and change in TL. Thus, further prospective birth cohort studies should be considered in this field. Fifth, adults who participated in the online mental health questionnaire tended to be healthier, better educated, and had higher socioeconomic status compared with all UKB participants and the general UK residents in the same age range.^[^ ^47^ ^]^ Thus, our findings may not be generalizable to the general population in the UKB or other population in the UK as there may be “healthy volunteer” selection bias.^[^ ^48^ ^]^

In this large lifespan analysis of middle‐aged and older adults in the UKB, we found a sex‐specific association between childhood adversity and shorter TL; women with childhood adversity were more likely to have shorter TL compared to men. Furthermore, unhealthy lifestyle and mental disorder partially mediated the associations in women, especially for physical and sexual abuse. These findings highlighted the importance of behavioral and psychological interventions in promoting healthy aging among women who experienced childhood adversity.

## 4 Experimental Section

### Study Population

**Figure 4:**
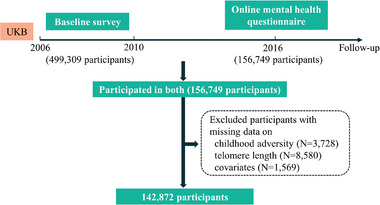
Flow chart of analytic sample.

UKB is a large population‐based cohort study conducted from 2006 to 2010 and recruited ≈500 000 participants aged 40–69 years. The data on the baseline questionnaire and anthropometric measures were collected at 22 assessment centers across England, Wales, and Scotland. A detailed description of the cohort study was published elsewhere.^[^ ^48^, ^49^ ^]^ In 2016, one‐third of the participants (N = 156749) responded to the online mental health questionnaire. Participants with missing data on childhood adversity (N = 3728), TL (N = 8580), or covariates (N = 1569) were excluded, resulting in 142872 participants included in the analyses (**Figure** **4**). The UKB study was approved by the North West Multi‐Centre Research Ethics Committee as a Research Tissue Bank (21/NW/0157). Written informed consent was provided by each participant before joining this study. The Strengthening the Reporting of Observational Studies in Epidemiology reporting guidelines was followed.

### Childhood Adversity

Childhood adversity, including physical neglect, emotional neglect, sexual abuse, physical abuse, and emotional abuse, was sourced from the 2016 online mental health questionnaire with the Childhood Trauma Screener, a validated screening tool^[^ ^50^ ^]^: 1) someone to take to doctor when needed as a child (physical neglect); 2) felt loved as a child (emotional neglect); 3) sexually molested as a child (sexual abuse); 4) physically abused by family as a child (physical abuse); or 5) felt hated by a family member (emotional abuse).^[^ ^51^ ^]^ Each individual type was referred to as, “When I was growing up (age <16 years old)”. The response to every question contains never true, rarely true, sometimes true, often true, and very often true. Each individual type was scored 0 or 1 according to the cutoff points derived from validation studies.^[^ ^50^, ^52^ ^]^ Physical neglect was assigned as 1 if participants reported never true, rarely true, sometimes true, or often true; emotional neglect was assigned as 1 if participants reported never true, rarely true, or sometimes true; sexual abuse, physical abuse, and emotional abuse was assigned as 1 if participants reported rarely true, sometimes true, often true, and very often true. Thus, the cumulative score ranged from 0 to 5, with a higher score indicating more frequent childhood adversities. The details of questions and cutoff points are shown in Table S8 (Supporting Information).

### Leucocyte TL

Leucocyte TL at the baseline survey was measured by quantitative PCR, a widely accepted and well‐validated technique.^[^ ^53^, ^54^ ^]^ This measure (Data‐field: 22 191) is a ratio (T/S) of telomere repeat copy number (T) to single copy gene copy number (S), relative to a standard sample, and was further adjusted for the influence of technical parameters (i.e., enzyme, PCR machine, primer, operator, temperature and DNA purity). Strict and pre‐defined quality control (QC) criteria were applied for the measurement of TL at both the sample and run levels. Samples failed QC if they met any of the listed criteria: contamination, inaccurate calibration or pipetting, incorrect master mix preparation, potential sample quality issues, and insufficient DNA quantity/inaccurate sample dilution. All samples that failed QC were re‐measured until valid measurements were obtained or the sample was considered exhausted or unsatisfactory. Besides, deliberate repeats and blinded duplicates were applied to measure reproducibility and stability of the measurement. A detailed description of the measurement and QC procedure of TL in the UKB participants was published elsewhere.^[^ ^55^ ^]^

### Unhealthy Lifestyle Index

As done in previous studies,^[^ ^56^ ^]^ an unhealthy lifestyle index at the baseline survey was measured by five components (i.e., body mass index [BMI], physical activity, drinking, smoking, and diet) collected by structured questionnaires and 24‐h dietary recall. The unhealthy level of each component was scored 1 and otherwise, was scored 0. The unhealthy lifestyle index ranged from 0 to 5, with a higher score indicating a higher level of unhealthy lifestyle (see details in Methods S1, Supporting Information).

### Mental Disorder Index

Mental disorder index at the baseline survey was defined using the following disorders: depression, assessed using an ICD‐9/10 code of primary or secondary diagnosis (Data‐fields: ICD‐9: 41 271 (311); ICD‐10: 41 270 (F32‐F33)) and self‐reported depression (Data‐field: 20 002 (1286, 1531))^[^ ^57^ ^]^; anxiety, assessed using an ICD‐9/10 code (Data‐fields: ICD‐9: 41 271 (300); ICD‐10: 41 270 (F40‐F48)) and self‐reported anxiety (Data‐field: 20 002 (1287))^[^ ^58^ ^]^; insomnia (Data‐field:1200), obtained by asking “Do you have trouble falling asleep at night or do you wake up in the middle of the night?” (usually vs sometimes or rarely/never).^[^ ^59^ ^]^ Participants with each disorder was scored 1 and otherwise, was scored 0. The mental disorder index ranged from 0 to 3, with a higher score indicating a higher level of mental disorder.

### Covariates

Covariates collected at baseline through a questionnaire included age, sex, race and ethnicity, occupation, educational level, TDI, and history of cardiovascular disease (CVD) and cancer (see details in Methods S2, Supporting Information).

PRS for TL was constructed using seven SNPs selected from a meta‐analysis of genome‐wide association studies in 37 684 people of European ancestry (data from UKB was not included) (Table S9, Supporting Information).^[^ ^6^ ^]^ According to the number of TL associated alleles, the genetic variants were recoded to 0, 1, or 2. The seven variants were weighted by the effect size (β coefficient) to create a PRS for TL for each participant:

(1) $$\mathrm{PRS}\;\mathrm{for}\;\mathrm{TL}\;=\;\beta_{1}\times \mathrm{SN}P_{1\;}+\;\beta_{2}\times \mathrm{SN}P_{2}+\cdots \cdots \beta_{n}\times \mathrm{SN}P_{n}$$

### Statistical Analyses

The basic characteristics of the participants were described with means ± SD for continuous variables and numbers (percentage) for categorical variables. The differences in characteristics between different sex were compared using t‐test for continuous variables and χ^2^ test for categorical variables. Multiple imputations were used by chained equations with five copies of imputation^[^ ^60^ ^]^ to impute missing values on BMI (N = 293), physical activity (N = 927), smoking (N = 1757), diet (N = 2), drinking (N = 17 578).

The association between childhood adversity and TL in all participants was estimated using general linear regression models, and the coefficients and corresponding 95% CIs were documented from three models. Model 1 was adjusted for age and sex. Model 2 was additionally adjusted for race and ethnicity, occupation, educational level, TDI, and history of CVD and cancer. Model 3 was further adjusted for PRS based on model 2 given that TL was partly determined by genetic susceptibility. Second, to estimate the sex differences in the association, the stratification analyses was performed by sex. A cross‐product term was also added in model 3 to examine the multiplication interaction of childhood adversity and sex on TL.

Third, considering only significant associations of childhood adversity with TL in women were observed, further examination was conducted to assess whether unhealthy lifestyles and mental disorders mediated the associations in women by performing the following analyses. First, general linear regression models were used to examine the associations of childhood adversity with unhealthy lifestyle index and mental disorder index, respectively. Similar linear regression models were used to examine the associations of unhealthy lifestyle index and mental disorder index with TL. Model 1 was adjusted for age. Model 2 was further adjusted for race and ethnicity, occupation, educational level, TDI, and history of CVD and cancer. Next, the R package “mediation” with 1000 simulations was used to perform the mediation analysis. The proportions of indirect effects and corresponding 95% CIs were documented after adjustment for age. The proportions of indirect effects were calculated using average causal indirect effects/ (average causal indirect effects +average direct effects).

To test the robustness of the findings, first, the basic characteristics were compared between included participants and participants excluded from this study due to missing data. Then, the mediation analysis was repeated using a complete‐case sample.

For replication, 5282 participants were used in the US HRS to test the associations between childhood adversity and TL in men and women. General linear regression models were used. Covariates collected in 2008 included age, sex, race and ethnicity, educational level, marital status, history of smoking, BMI, and number of medical conditions ever reported (diabetes, high blood pressure, heart disease, lung disease, cancer, stroke, arthritis, and psychiatric problems) (see details in Methods S3, Supporting Information).

All analyses were conducted using SAS version 9.4 (SAS Institute, Cary, NC) and R 4.1.3 (http://www.R‐project.org). Two‐tailed *P* value < 0.05 was considered statistically significant.

## Conflict of Interest

The authors declare no conflict of interest.

## Author Contributions

J.Y. and F.P. contributed equally to this work as co‐first author. Z.L. and X.W. designed and supervised the study. J.Y. and F.P. analyzed the data and drafted the manuscript. J.Y., F.P., G.Y., X.C., J.Z., and Z.L. interpreted the results. G.Y., X.C., J.Z., M.H., H.Z., L.Z., Y.W., X.W., and Z.L. revised the manuscript. Z.L. and X.W. took responsibility for the content of the article. All authors have read and approved the submitted version of the manuscript.

## Supporting information

Supporting Information

## Data Availability

The data that support the findings of this study are openly available in UK Biobank at [https://www.ukbiobank.ac.uk/], reference number [61856].
