# Supplementary material for: Sex‐Specific Association Between Childhood Adversity and Accelerated Biological Aging
Source: Adv Sci (Weinh). 2024 May 5;11(26):2309346. doi: 10.1002/advs.202309346 (PMC11234451; doi:10.1002/advs.202309346)
Supplement: Supplementary file 1 — Supporting Information [file ADVS-11-2309346-s001.pdf]

ADVANCED SCIENCE
Open Access

Supporting Information

for *Adv. Sci.*, DOI 10.1002/advs.202309346

Sex-Specific Association Between Childhood Adversity and Accelerated Biological Aging

Jie Yu, Fan Pu, Gan Yang, Meng Hao, Hui Zhang, Jingyun Zhang, Xingqi Cao, Lijun Zhu, Yuhui Wan, Xiaofeng Wang and Zuyun Liu**

Supporting Information

Sex-specific Association between Childhood Adversity and Accelerated Biological Aging

Jie Yu[#], *Fan Pu*[#], *Gan Yang, Meng Hao, Hui Zhang, Jingyun Zhang, Xingqi Cao, Lijun Zhu, Yuhui Wan, Xiaofeng Wang*[*], *Zuyun Liu*[*]

***Corresponding authors:**

Zuyun Liu, Professor, Center for Clinical Big Data and Analytics Second Affiliated Hospital and Department of Big Data in Health Science School of Public Health, The Key Laboratory of Intelligent Preventive Medicine of Zhejiang Province, Zhejiang University School of Medicine, 866 Yuhangtang Rd, Hangzhou 310058, Zhejiang, China. Telephone: +86-0571-87077127.

Email: Zuyun.liu@outlook.com or zuyunliu@zju.edu.cn

Xiaofeng Wang, Professor, Human Phenome Institute, National Clinical Research Center for Ageing and Medicine, Huashan Hospital, and State Key Laboratory of Genetic Engineering, Collaborative Innovation Center for Genetics and Development, School of Life Sciences, Fudan University, Shanghai 200433, China.

Email: wangxiaofeng@fudan.edu.cn

Supplementary Information Text

Methods S1. Assessment of lifestyle

Methods S2. Assessment of covariates

Methods S3. Methods in the US Health and Retirement Study (HRS)

Table S1. Associations of childhood adversity with unhealthy lifestyle index in women

Table S2. Associations of childhood adversity with mental disorder index in women

Table S3. Associations of unhealthy lifestyle index with telomere length in women

Table S4. Associations of mental disorder index with telomere length in women

Table S5. Comparison of characteristics between included participants and those excluded because of missing data

Table S6. Proportions of indirect effects of childhood adversity in telomere length attributed to unhealthy lifestyle index and mental disorder index in women in a complete-case sample (N=66,492)

Table S7. Characteristics of the study participants in HRS

Table S8. Questions and responses for variables included in the childhood adversity in UK biobank

Table S9. Telomere length associated SNPs and effect sizes on telomere length

Methods S1. Assessment of lifestyle

According to the World Health Organization recommendations,[1] we assigned a point of 1 to participants whose BMI was out of the range of 18.5-24.9 kg/m^2. For smoking, we assigned a point of 1 to participants who smoked more than 100 cigarettes in a lifetime.[2] For drinking, we assigned a point of 1 to men who consumed alcohol two drinks or more every day and to women who consumed alcohol one drink or more every

day.[2] For physical activity, we assigned a point of 1 to participants who engaged in vigorous activity less than 75 minutes or once per week or engaged in moderate physical activity less than 150 minutes or 5 days per week.[3] For diet, we assigned a point of 1 to participants who had not achieved the intake goals (inadequate intake or excessive intake) for more than half of the following components: fruits, vegetables, dairy products, (shell)fish, whole grains, vegetable oils, refined grains, processed meats, and sugar-sweetened beverages.[4] Details of the intake goals of each diet component have been described elsewhere.[4,5]

Methods S2. Assessment of covariates

Race and ethnicity were collected due to their potential confounding and were categorized as Black, Chinese, White, South Asian, multiple ethnicities, and other (i.e., any other race and ethnicity not already specified).[6] Occupation was categorized as working, retired, and other (full-time or part-time student, unpaid or voluntary work, unemployed, unable to work because of sickness or disability, looking after home and/or family, or did not answer).[6] Educational level was categorized as high (college or university degree), intermediate (A/AS levels or O-levels, general certificate of secondary education, or equivalent), and low (none of the above).[2] Census data on employment, social class, and housing based on the postal code of participants was used for Townsend Deprivation Index (TDI).[6] A higher TDI denotes a lower area level of socioeconomic status. History of disease (i.e., cardiovascular disease and cancer) was considered in this study, which was categorized as yes or no.

Methods S3. Methods in the US Health and Retirement Study (HRS)

The HRS is a longitudinal, nationally representative sample including 26,000 US residents older than 50 years of age and their spouses.[7] Telomere length was assayed using qPCR from 5,808 participants who provided a saliva sample in 2008.[8] Measures of childhood adversity, including physical abuse and emotional neglect, were collected in survey years 2006 - 2012. Physical abuse was obtained by asking ‘Before you

were 18 years old, were you ever physically abused by either of your parents?' (yes vs. no). Emotional neglect was obtained by asking 'Before you were 18 years old, how much time and attention did your mother give you when you needed it?' (a little or not at all vs. a lot or some). Covariates were collected in 2008, including age, sex, race and ethnicity (non-Hispanic white, non-Hispanic black, Hispanic, and other), education level (high school and above, less than high school), marital status (married or partnered, other), smoking status (never smoked, ever smoked), BMI (underweight, normal, overweight, obese), and number of medical conditions (zero, one, two, three, and four or more). Medical conditions included diabetes, high blood pressure, heart disease, lung disease, cancer, stroke, arthritis and psychiatric problems.[9]

Table S1. Associations of childhood adversity with unhealthy lifestyle index in women

Childhood adversity	β (95%CI)	
	**Model 1[a]**	**Model 2[b]**
Physical neglect	0.070 (0.048, 0.092)	0.033 (0.011, 0.054)
Emotional neglect	0.178 (0.158, 0.197)	0.153 (0.133, 0.172)
Sexual abuse	0.206 (0.180, 0.232)	0.183 (0.158, 0.209)
Physical abuse	0.213 (0.192, 0.235)	0.194 (0.172, 0.215)
Emotional abuse	0.198 (0.177, 0.220)	0.176 (0.155, 0.198)
Cumulative score	0.088 (0.081, 0.094)	0.075 (0.068, 0.082)

Abbreviation: CI, confidence interval.

[a)] Model 1: adjusted for age.

[b)] Model 2: further adjusted for race and ethnicity, educational level, occupation, Townsend deprivation index and history of cardiovascular disease and cancer based on Model 1.

Table S2. Associations of childhood adversity with mental disorder index in women

Childhood adversity	β (95%CI)	
	**Model 1[a]**	**Model 2[b]**
Physical neglect	0.077 (0.067, 0.087)	0.067 (0.057, 0.078)
Emotional neglect	0.150 (0.140, 0.159)	0.144 (0.135, 0.154)

Sexual abuse	0.109 (0.096, 0.121)	0.105 (0.093, 0.117)
Physical abuse	0.082 (0.072, 0.093)	0.079 (0.068, 0.089)
Emotional abuse	0.134 (0.124, 0.144)	0.130 (0.119, 0.140)
Cumulative score	0.057 (0.054, 0.061)	0.055 (0.052, 0.059)

Abbreviation: CI, confidence interval.

[a)] Model 1: adjusted for age.

[b)] Model 2: further adjusted for race and ethnicity, educational level, occupation, Townsend deprivation index and history of cardiovascular disease and cancer based on Model 1.

Table S3. Association of unhealthy lifestyle index with telomere length in women

	β (95%CI)	
	**Model 1[a]**	**Model 2[b]**
Unhealthy lifestyle index	-0.0028 (-0.0036, -0.0020)	-0.0024 (-0.0032, -0.0016)

Abbreviation: CI, confidence interval.

[a)] Model 1: adjusted for age.

[b)] Model 2: further adjusted for race and ethnicity, educational level, occupation, Townsend deprivation index and history of cardiovascular disease and cancer based on Model 1.

Table S4. Association of mental disorder index with telomere length in women

	β (95%CI)	
	**Model 1[a]**	**Model 2[b]**
Mental disorder index	-0.0032 (-0.0049, -0.0016)	-0.0025 (-0.0042, -0.0009)

Abbreviation: CI, confidence interval.

[a)] Model 1: adjusted for age.

[b)] Model 2: further adjusted for race and ethnicity, educational level, occupation, Townsend deprivation index and history of cardiovascular disease and cancer based on Model 1.

Table S5. Comparison of characteristics between included participants and those excluded because of missing data

	Total (N=153021)	Included (N=142872)	Excluded (N=10149)	*P* value
Age, mean (SD), y	56.4 (7.7)	56.4 (7.7)	56.4 (7.7)	0.979
Sex, women	86204 (56.3)	80298 (56.2)	5906 (58.2)	<.001
Race and ethnicity				<.001
White	148242 (97.2)	138945 (97.3)	9297 (96.1)	
Chinese	349 (0.2)	323 (0.2)	26 (0.3)	
South Asian	1259 (0.8)	1146 (0.8)	113 (1.2)	
Black	1065 (0.7)	948 (0.7)	117 (1.2)	
Multiple	794 (0.5)	733 (0.5)	61 (0.6)	
Other[a]	837 (0.5)	777 (0.5)	60 (0.6)	
Educational level[b]				0.070
High	70591 (46.4)	66134 (46.3)	4457 (47.5)	
Intermediate	50068 (32.9)	47064 (32.9)	3004 (32.0)	
Low	31600 (20.8)	29674 (20.8)	1926 (20.5)	
Occupation				0.043
Working	97503 (63.9)	91154 (63.8)	6349 (64.6)	
Retired	45077 (29.5)	42278 (29.6)	2799 (28.5)	
Other[c]	10122 (6.6)	9440 (6.6)	682 (6.9)	
Townsend deprivation index, mean (SD)	-1.7 (2.8)	-1.7 (2.8)	-1.6 (2.9)	<.001
Depression, yes	8076 (5.3)	7537 (5.3)	539 (5.3)	0.877
Anxiety, yes	2263 (1.5)	2119 (1.5)	144 (1.4)	0.604
Insomnia, yes	39924 (26.1)	37262 (26.1)	2662 (26.2)	0.742
BMI, mean (SD), kg/m^2	26.8 (4.6)	26.8 (4.6)	26.8 (4.6)	0.516
≤18.5	857 (0.6)	793 (0.6)	64 (0.6)	
18.5-24.9	58477 (38.3)	54648 (38.3)	3829 (38.0)	
≥24.9	93309 (61.1)	87138 (61.1)	6171 (61.3)	
Smoking, yes	63992 (41.9)	59974 (42.0)	4018 (41.4)	0.288

Drinking, yes	60349 (39.8)	56844 (39.8)	3505 (39.8)	0.963
Irregular exercise, yes	39400 (25.8)	36746 (25.7)	2654 (26.6)	0.046
Unhealthy diet, yes	94102 (61.5)	87725 (61.4)	6377 (63.3)	<.001
Prevalent CVD, yes	6733 (4.4)	6291 (4.4)	442 (4.4)	0.819
Prevalent cancer, yes	12008 (7.8)	11086 (7.8)	922 (9.1)	<.001
Polygenic risk score for telomere length, mean (SD)	-0.42 (0.11)	-0.42 (0.11)	-0.42 (0.11)	0.222
Telomere length, mean (SD)	0.84 (0.13)	0.84 (0.13)	0.83 (0.13)	0.122

Abbreviation: BMI, body mass index; CVD, cardiovascular disease.

[a)] Other includes any races or ethnicities not otherwise specified.

[b)] High educational level: college or university degree; Intermediate educational level: A/AS levels or equivalent, O levels/GCSEs or equivalent; Low educational level: none of the aforementioned.

[c)] Other includes full-time or part-time student, unpaid or voluntary work, unemployed, unable to work because of sickness or disability, looking after home and/or family, and did not answer.

Table S6. Proportions of indirect effects of childhood adversity in telomere length attributed to unhealthy lifestyle index and mental disorder index in women in a complete-case sample (N=66,492)[a]

Childhood adversity	Unhealthy lifestyle index		Mental disorder index	
	**Proportions of indirect effects (%), 95%CI**	***P* value**	**Proportions of indirect effects (%), 95%CI**	***P* value**
Physical neglect	4.1 (1.8, 15.0)	0.008	6.2 (2.3, 22.0)	0.008
Emotional neglect	23.5 (-257.4, 177.0)	0.150	24.8 (-255.2, 195.0)	0.140
Sexual abuse	14.8 (6.3, 81.0)	0.028	9.7 (3.1, 56.0)	0.028
Physical abuse	12.0 (6.7, 29.0)	0.002	5.7 (2.2, 15.0)	<.001
Emotional abuse	29.5 (-353.2, 374.0)	0.300	24.9 (-305.3, 347.0)	0.300
Cumulative score	15.1 (8.5, 36.0)	0.002	11.9 (4.4, 31.0)	0.002

Abbreviation: CI, confidence interval.

a) All models were adjusted for age.

Table S7. Characteristics of the study participants in HRS

Variables	Total (N=5282)	Female (N=3106)	Male (N=2176)	*P* Value
Age, mean (SD), y	69.3 (10.2)	68.8 (10.5)	70.0 (9.6)	<.001
Race and ethnicity				0.010
Non-Hispanic white	3815 (78.3)	2318 (74.6)	1705 (78.4)	
Non-Hispanic black	558 (11.4)	427 (13.7)	240 (11.0)	
Hispanic	438 (9.0)	316 (10.2)	198 (9.1)	
Other[a]	63 (1.3)	45 (1.4)	33 (1.5)	
Marital status				<.001
Married/partnered	3299 (67.7)	1778 (57.2)	1753 (80.6)	
Other	1575 (32.3)	1328 (42.8)	423 (19.4)	
Educational level				0.918
High school and above	3959 (81.2)	2495 (80.4)	1751 (80.5)	
Less than high school	915 (18.8)	610 (19.6)	425 (19.5)	
BMI				<.001
Normal	1378 (28.3)	964 (31.0)	536 (24.6)	
Underweight	59 (1.2)	57 (1.8)	9 (0.4)	
Overweight	1848 (37.9)	1036 (33.4)	952 (43.8)	
Obese	1589 (32.6)	1049 (33.8)	679 (31.2)	
Smoking status				<.001
Never smoked	2114 (43.4)	1587 (51.1)	687 (31.6)	

Ever smoked	2760 (56.6)	1519 (48.9)	1489 (68.4)	
Number of medical conditions				0.136
0	584 (12.0)	350 (11.3)	276 (12.7)	
1	1091 (22.4)	728 (23.4)	451 (20.7)	
2	1293 (26.5)	828 (26.7)	579 (26.6)	
3	1047 (21.5)	653 (21.0)	477 (21.9)	
4 or more	859 (17.6)	547 (17.6)	393 (18.1)	
Physical abuse, yes	350 (7.2)	268 (8.6)	114 (5.2)	<.001
Time/attention from mother				<.001
A little/not at all	608 (12.5)	2596 (83.6)	1952 (89.7)	
A lot/some	4266 (87.5)	510 (16.4)	224 (10.3)	
Telomere length, mean (SD)	1.38 (0.73)	1.38 (0.68)	1.37 (0.79)	0.685

Abbreviation: BMI, body mass index.
[a] Other includes any races or ethnicities not otherwise specified.

Table S8. Questions and responses for variables included in the childhood adversity in UK biobank

**Childhood traumas questions**		**Responses/description**	**Cut off**
Physical neglect	Someone to take to doctor when needed as a child.	0, Never true; 1, Rarely true; 2, Sometimes true; 3, Often; 4, Very often true	≤3
Emotional neglect	Felt loved as a child.	0, Never true; 1, Rarely true; 2, Sometimes true; 3, Often; 4, Very often true	≤2
Sexual abuse	Sexually molested as a child.	0, Never true; 1, Rarely true; 2, Sometimes true; 3, Often; 4, Very often true	≥1
Physical abuse	Physically abused by family as a child.	0, Never true; 1, Rarely true; 2, Sometimes true; 3, Often; 4,	≥1

		Very often true	
Emotional abuse	Felt hated by family member as a child.	0, Never true; 1, Rarely true; 2, Sometimes true; 3, Often; 4, Very often true	≥1
Cumulative score	Summary score of childhood adversity	Summary score of five childhood adversity items (0-5).	—

Table S9. Telomere length associated SNPs and effect sizes on telomere length

SNP	Chr	Position	Gene	Effect Allele	β	Std Error
rs10936599	3	170974795	TERC	T	-0.097	0.008
rs2736100	5	1339516	TERT	A	-0.078	0.009
rs7675998	4	164227270	NAF1	A	-0.074	0.009
rs9420907	10	105666455	OBFC1	A	-0.069	0.010
rs8105767	19	22007281	ZNF208	A	-0.048	0.008
rs755017	20	61892066	RTEL1	A	-0.062	0.011
rs11125529	2	54329370	ACYP2	C	-0.056	0.010

SI Reference

[1] Obesity: preventing and managing the global epidemic. Report of a WHO consultation. *World Health Organ Tech Rep Ser* **2000**, *894*, i.

[2] Y. B. Zhang, C. Chen, X. F. Pan, J. Guo, Y. Li, O. H. Franco, G. Liu, A. Pan, Associations of healthy lifestyle and socioeconomic status with mortality and incident cardiovascular disease: two prospective cohort studies. *Bmj* **2021**, *373*, n604.

[3] I. Lourida, E. Hannon, T. J. Littlejohns, K. M. Langa, E. Hyppönen, E. Kuzma, D. J. Llewellyn, Association of Lifestyle and Genetic Risk With Incidence of Dementia. *Jama* **2019**, *322*, 430.

[4] D. Mozaffarian, Dietary and Policy Priorities for Cardiovascular Disease, Diabetes, and Obesity: A Comprehensive Review. *Circulation* **2016**, *133*, 187.

[5] M. A. Said, N. Verweij, P. van der Harst, Associations of Combined Genetic and Lifestyle Risks With Incident Cardiovascular Disease and Diabetes in the UK Biobank Study. *JAMA Cardiol* **2018**, *3*, 693.

[6] Y. V. Chudasama, K. Khunti, C. L. Gillies, N. N. Dhalwani, M. J. Davies, T. Yates, F. Zaccardi, Healthy lifestyle and life expectancy in people with multimorbidity in the UK Biobank: A longitudinal cohort study. *PLoS Med* **2020**, *17*, e1003332.

[7] A. Sonnega, J. D. Faul, M. B. Ofstedal, K. M. Langa, J. W. Phillips, D. R. Weir, Cohort Profile: the Health and Retirement Study (HRS). *Int J Epidemiol* **2014**, *43*, 576.

[8] G. H. Steven, J. Connor. Technical Description of the Health and Retirement Study Sample Design. Ann Arbor, Michigan: Institute for Social Research, University of Michigan, 1995.

[9] E. Puterman, A. Gemmill, D. Karasek, D. Weir, N. E. Adler, A. A. Prather, E. S. Epel, Lifespan adversity and later adulthood telomere length in the nationally representative US Health and Retirement Study. *Proc Natl Acad Sci U S A* **2016**, *113*, E6335.
